# Th17/Treg balance: the bloom and wane in the pathophysiology of sepsis

**DOI:** 10.3389/fimmu.2024.1356869

**Published:** 2024-03-15

**Authors:** Xinyong Liu, Longwang Chen, Wei Peng, Hongsheng Deng, Hongying Ni, Hongjie Tong, Hangbo Hu, Shengchao Wang, Jin Qian, Andong Liang, Kun Chen

**Affiliations:** ^1^ Department of Critical Care Medicine, Affiliated Jinhua Hospital, Zhejiang University School of Medicine, Jinhua, China; ^2^ Emergency Department, The First Affiliated Hospital of Wenzhou Medical University, Wenzhou, China; ^3^ Nursing Faculty, School of Medicine, Jinhua Polytechnic, Jinhua, China

**Keywords:** sepsis, Th17, Treg, balance, pathophysiology

## Abstract

Sepsis is a multi-organ dysfunction characterized by an unregulated host response to infection. It is associated with high morbidity, rapid disease progression, and high mortality. Current therapies mainly focus on symptomatic treatment, such as blood volume supplementation and antibiotic use, but their effectiveness is limited. Th17/Treg balance, based on its inflammatory property, plays a crucial role in determining the direction of the inflammatory response and the regression of organ damage in sepsis patients. This review provides a summary of the changes in T-helper (Th) 17 cell and regulatory T (Treg) cell differentiation and function during sepsis, the heterogeneity of Th17/Treg balance in the inflammatory response, and the relationship between Th17/Treg balance and organ damage. Th17/Treg balance exerts significant control over the bloom and wanes in host inflammatory response throughout sepsis.

## Introduction

Sepsis is defined as an initial hyperinflammatory response to systemic infection caused by an invasive pathogen (1, 2). This response involves hemodynamic changes and leads to subsequent immunosuppression and organ dysfunction, eventually triggering multiple organ failure, secondary infection, and mortality (1, 2). According to data from Germany, approximately 48.9 million people suffer from sepsis each year, with sepsis-related deaths accounting for about 20% of all deaths (3, 4). A global observational study in 2017 recorded 11 million sepsis-related deaths, accounting for 19.7% of the total mortality rate for that specific year (5). In mainland China, sepsis patients make up a significant proportion of intensive care unit (ICU) admissions, with a 90-day mortality rate of 35.5%, imposing a substantial economic burden (6). From 1990 to 2017, the incidence of sepsis decreased by 37.0% and the mortality by 52.8% (5). Despite extensive investigations into the pathophysiology of sepsis in recent years, which have made substantial contributions to drug development and organ support therapy, sepsis continues to prevail as the primary driver of mortality in critically ill patients (7, 8).

Sepsis presents an extremely heterogeneous feature, due to its complex types of pathogen infections and differential organismal response ability (9). The inflammatory and immunosuppressive states of sepsis occur concurrently (10). In the early stages of sepsis, besides the fact that Toll-like receptor (TLR) located on the cell membrane surfaces of antigen-presenting cells (APC) (e.g., macrophage, dendritic cell (DC), and neutrophil) recognize either pathogen-associated molecular marker (PAMP) or damage-associated molecular patterns (DAMP) for initiation of inflammation, a massive decrease in T cells via apoptosis has been observed (11, 12). During the initial exposure phase to severe infection, TLR recognizes and binds PAMP, activating the innate immune system and complement systems (13). Then, large amounts of complement component 3a (C3a) and complement component 5a (C5a) are produced and secreted, prompting the release of pro-inflammatory cytokines, thereby triggering a cytokine storm (13). A prolonged hyperinflammatory response triggers cellular damage and promotes the release of DAMP, further bolstering the production and removal of inflammatory mediators (13). In addition, the hyperinflammatory response provokes coagulation and endothelial cell activation, resulting in disseminated intravascular coagulation and endothelial leakage (14, 15). Subsequently, the adaptive immune system takes effect, and the T cell receptor (TCR) activates T helper cells (Th, e.g., Th1, Th17) and cytotoxic T cells, releasing inflammatory factors and amplifying the inflammatory response to facilitate the clearance of the infection (16). Tregs have the opposite function to Th17, suppressing excessive T-cell responses and promoting self-tolerance under inflammatory conditions (17). The balance between Th subsets and Tregs is important for clearing the infection. When a compensatory anti-inflammatory response fails to balance an explosive pro-inflammatory response, sepsis is exacerbated and secondary infections are triggered, leading to organ dysfunction and death. Furthermore, in the distant phase of sepsis, the continued expression of adaptive anti-inflammatory markers alters the function of innate immune cells and induces lymphocyte exhaustion, which leads to prolonged immunosuppression (14, 18). This phase can last for several years (19).

**Figure 1:**
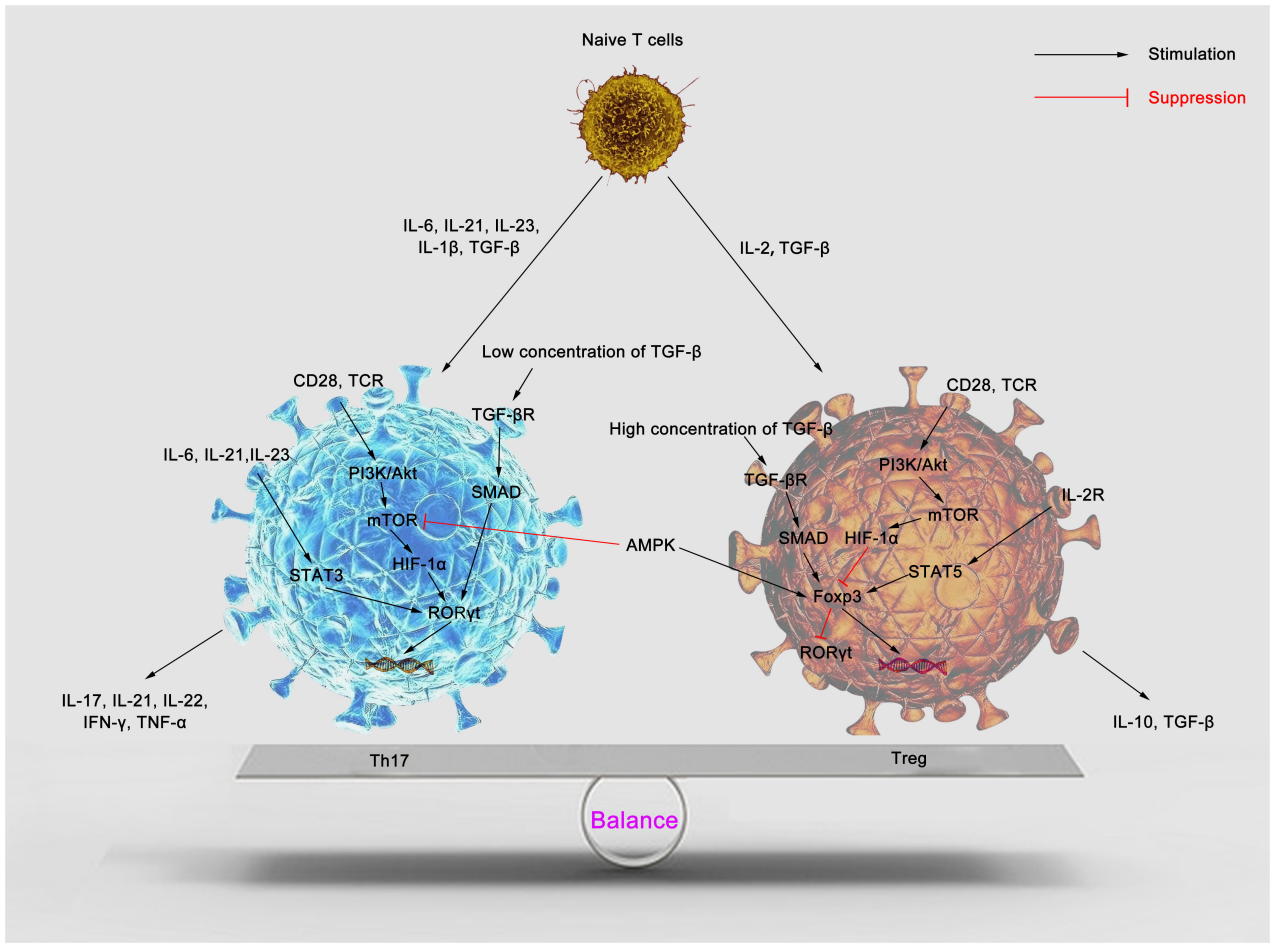
Cytokines regulate Th17/Treg balance. Naïve T cells can differentiate into Th17 and Tregs in response to specific cytokines. IL-1β, IL-6, IL-21, IL-23 and TGF-β induce Th17 differentiation. IL-6, IL-21 and IL-23 promote STAT3 phosphorylation, induce RORγt gene expression, and then stimulate CD4^+^ T cells to differentiate into Th17. mTOR is activated through the PI3K/Akt pathway to promote Th17 differentiation. A low TGF-β concentration activates SMAD and synergizes with IL-6 to promote Th17 production. Numerous mature Th17 secrete pro-inflammatory factors such as IL-17, IL-21, IL-22, IFN-γ, TNF-α, etc., promoting inflammation. Treg differentiation is mediated by IL-2 and TGF-β. IL-2/STAT5 pathway and high concentrations of TGF-β promote the expression of the Foxp3 gene and contribute to Treg differentiation. PI3K/Akt/mTOR pathway inhibits the expression of Foxp3 to suppress Treg differentiation. AMPK inhibits mTOR activity and promotes Foxp3 expression, which facilitates Treg differentiation. Many Tregs secrete IL-10 and TGF-β and induce host anti-inflammatory responses. Th17, T helper 17; Treg, regulatory T; IL, interleukin; TGF-β, transforming growth factor-β; TCR, T cell receptor; PI3K, phosphatidylinositol 3-kinase; Akt, protein kinase B; STAT3, signal transducer and activator of transcription 3; STAT5, signal transducer and activator of transcription 5; mTOR, mammalian target of rapamycin; RORγt, retinoid acid-related orphan receptor γt; HIF-1α, hypoxia inducible factor 1α; IFN-γ, interferon-γ; TNF-α, tumor necrosis factor-α; Foxp3, forkhead box protein P3; AMPK, AMP-activated protein kinase.

Th17 and regulatory T (Treg) cells are two subsets of CD4^+^ T cells that participate in the immune response through the defense, immune surveillance and immune regulation, and act to promote or suppress the inflammatory response (20). Th17 are differentiated mainly from T cells stimulated by transforming growth factor-β (TGF-β) (21), IL-6 (22), IL-21 (23), IL-23 (24) and IL-1β (25), which further secrete IL-17 and various inflammatory factors, such as IL-6, IL-22, IL-23, granulocyte-colony stimulating factor (GCSF) and tumor necrosis factor-alpha (TNF-α), to induce recruitment of neutrophils (26–28). Forkhead box protein 3 (Foxp3), a transcription factor, is involved in the Tregs differentiation. Tregs mainly secret IL-10 and TGF-β to maintain self-tolerance and suppress inflammation (29–31). In sepsis, Foxp3 inhibits T-cell function, while IL-17 promotes its function, and the two thereby oppose and interact with each other (32–35). As such, during the inflammatory response, the alterations in the function and quantity of Th17 and Tregs can effectively modulate the immune response (**Figure 1**). In sepsis, the altered immune function of T lymphocytes is an essential driver of increased mortality and poor prognosis (36). A study has confirmed that Th17, a central component in inflammatory diseases, is significantly elevated in septic patients, which correlates with the intensive inflammatory response and severity of the disease (37). Meanwhile, some experimental and clinical trials have shown that sepsis enhances Treg function, which acts on the innate and adaptive immune system, suppressing immune function and leading to immune paralysis and, ultimately, septic death (38–40). Compared to survivors of sepsis, deceased patients have a higher Th17/Treg ratio in the circulation, shifting the balance toward Th17 (41). Restoration of the Th17/Treg balance affects the prognosis of the disease and contributes to the treatment of sepsis.

The immune response is designed to clear infectious agents, but the excessive pro-inflammatory response will lead to multiple organ damage (42–44). Meanwhile, the compensatory anti-inflammatory response, aimed at suppressing the excessive inflammation, can lead to immunosuppression (45). These two key events in the inflammatory response are interactive and concomitant (46). Th17 are pro-inflammatory, while Tregs are anti-inflammatory (47). In recent years, Th17/Treg balance has been increasingly recognized as critical in preventing excessive immune activation (48–51), autoimmune responses (52–54), and the pathogenesis of metabolic syndrome (55, 56). This review discusses the changes in Th17/Treg balance and its role in the pathophysiology of sepsis.

## Sepsis and inflammation

Treatment of sepsis is currently focused on continuous fluid resuscitation, organ support, and anti-infective therapy with antibiotics (57). To date, novel effective strategies that directly target the physiological mechanisms are not available (58). The essence of sepsis is an uncontrolled immune response eroded by the host in the fight against pathogens or stresses, leading to multi-organ dysfunction (59). Clinical and animal studies on sepsis still rely on the characterization of systemic inflammatory response syndromes (60–63). Numerous studies have confirmed that excessive inflammatory response and cytokine storm during sepsis are the leading causes of poor prognosis (64–66). Our previous study confirmed that the inflammatory response in septic mice was enhanced by inhibiting SUMOylation of peritoneal macrophages and increasing NF-κB p65 activity, inducing inflammatory storm, which markedly inhibited intra-abdominal bacterial clearance and greatly exacerbated organ damage and mortality (67). Another study, which enrolled 3,250 subjects, found that serum concentrations of IL-1β, TNF-α, and interferon (IFN)-γ were elevated in patients with sepsis (68). The mean TNF-α concentration in sepsis was increased approximately 10-fold compared to the mean concentration in healthy participants (68). Elevated TNF-α concentration was associated with increased 28-day sepsis mortality (68). Weber and Ebihara et al. also found that the levels of inflammatory factors IL-3, IL-6, and growth differentiation factor (GDF)-15 were significantly elevated in sepsis and correlated with prognosis (64, 69). Overexpression of inflammatory factors leads to an aggressive inflammatory response in the host. Failure to control the intensity of the inflammatory response promptly triggered a persistent and excessive inflammatory response, which severely compromised the prognosis of patients with sepsis. However, drugs targeting inflammatory mediators, like IL-1β, TNF-α, or TLR, to suppress inflammation are ineffective in improving survival in sepsis patients (70–72).

## Th17 and Treg differentiation and function in the sepsis

Th17 and Tregs antagonize each other during the inflammatory reaction and the immune response (73). Th17 produces many pro-inflammatory factors, which recruit neutrophils and promote inflammation. In contrast, Tregs produces anti-inflammatory factors that inhibit the activity of immune cells, thereby suppressing the inflammatory response to avoid overactivation of inflammation (74). Together, Th17 and Tregs direct the immune process of sepsis.

Th17 can be induced to differentiate by cytokines IL-1β, IL-6, IL-21, IL-23, and TGF-β (**Table 1**) (89). Signal transducer and activator of transcription 3 (STAT3) and SMAD are activated by cytokines, which in turn induce IL-17 and the transcription factor retinoic acid-related orphan receptor-γt (RORγt) to differentiate Th17 (90, 91). Th17 mediates inflammatory response and promotes host clearance of infected lesions (92). The main mechanisms include the secretion of pro-inflammatory cytokines (e.g., IL-17, IL-21, IL-22, IFN-γ), aggregation of neutrophils, activation of innate immune cells, and enhancement of B cell function (93–95). Various studies have shown that Th17 and the secreted cytokine IL-17 are inextricably linked to the pathogenesis of sepsis (35, 96–99). Mohammad et al. confirmed that sepsis led to upregulation of IL-2-inducible T-cell kinase (ITK) protein (35). ITK regulated the expression of transcription factors such as nuclear factor of activated T cells cytoplasmic 1 (NFATc1) and STAT3 in naïve CD4^+^ T cells (Th0), promoting their transformation into Th17 (96, 97). Inhibition of ITK expression using ITK inhibitors significantly reduced the number of IL-17A^+^CD4^+^ T cells, and decreased the levels of inflammatory factors IL-6 and monocyte chemotactic factor 1 (MCP-1) (35). That shows the involvement of Th17 in the inflammatory response in sepsis. In addition, overexpression of calcium release-activated calcium modulator 1 (Orai1) inhibited the growth of the Th17 population in sepsis and reduced mortality and organ damage in septic mice (98). Meanwhile, Li et al. have shown that the percentages of Th17 and the levels of IL-17, IL-22 were independent risk factors for septic lung injury (99). Th17 and the cytokines it releases are involved in the pathogenesis and prognosis of sepsis due to their pro-inflammatory properties.

**Table 1:** The role of cytokines in Th17/Treg balance.

Cytokines	Function	Mechanism	Th17	Treg	Th17/Treg
IL-1β (75)	Pro-inflammatory	Induces gammadelta T cells to produce innate IL-17 and enhance Th17 responses	↑		↑
IL-6 (76)	Pro-inflammatory	induces STAT3 phosphorylation, increases expression of RORγt and RORα and promotes Th17 differentiation by cooperating with TGF-β	↑		↑
IL-18 (77)	Pro-inflammatory	decreases levels of Foxp3, decreases Foxp3 di- and oligomerization, and increases the ubiquitination and proteasomal degradation of Foxp3 to decreased Treg function.		↓	↑
IL-21 (78)	Pro-inflammatory	promotes CD4^+^ T cells proliferation and Th17 differentiation, and inhibits Treg differentiation by upregulating RORγt expression and downregulating Foxp3 expression	↑	↓	↑
IL-23 (79)	Pro-inflammatory	promotes the stability and survival of Th17	↑		
IL-33 (80)	Pro-inflammatory	IL-33 deficiency activates and matures DC to secret IL-6 and IL-23, suppressing Th17 responses and regulating the Th17/Treg balance	↑		↑
HIF-1α (81)	Pro-inflammatory	Induces transcriptional activation of RORγt and forms a tertiary complex with RORvt to promote Th17 differentiation; attenuates Treg development by binding Foxp3 and targeting it for proteasomal degradation	↑	↓	↑
IL-2 (82, 83)	Anti-inflammatory	promotes STAT5 phosphorylation and induces Foxp3 transcription for Treg differentiation		↑	↓
IL-4 (84)	Anti-inflammatory	suppresses activation of STAT6 and activating trascription factor 3 (ATF3) to abrogates Th17	↓		↓
IL-10 (85)	Anti-inflammatory	IL-10 deficiency increased CD4^+^ T cells, CD8^+^ T cells and Th17 but not Treg cells	↓		↓
IL-15 (86, 87)	Anti-inflammatory	impaired delivery of IL-15 to CD4^+^ T cells downmodulates Foxp3 expression (diminishing STAT5 phosphorylation) and enhances RORγt expression	↓	↑	↓
TNF-α (88)	Anti-inflammatory	upregulates MHC-II on alveolar type II cells through CXCR-2 to contribute to Treg expansion		↑	↓
TGF-β (21)	Anti-inflammatory	Stimulates naïve T cell to produce SMAD2, SMAD3 and SMAD4, promotes Foxp3 expression and Tregs generation		↑	↓

Tregs contribute to the maintenance of immune homeostasis and immune tolerance (100). Tregs differentiate from naïve T cells in the thymus or peripheral lymphoid tissue to protect organisms from autoimmune diseases and promote tissue repair (**Table 1**) (101). The main mechanisms of Tregs include: (1) contact-dependent mechanism: expression of co-stimulatory receptors, including cytotoxic T lymphocyte-associated antigen-4 (CTLA-4), programmed cell death 1 (PD-1), T-cell immunoglobulin and mucin-domain containing-3 (TIM-3), T cell immunoglobulin and ITIM domain (TIGIT), lymphocyte-activation gene 3 (LAG-3), ICOS, GITR, to prevent activation and maturation of DC or to prevent activated T cell response (102–106); (2) contact-independent mechanism: secretion of anti-inflammatory factors (IL-10, IL-35, TGF-β) to inhibit T cell proliferation (21, 107, 108); (3) CD25/IL-2 depletion mechanism: CD25, as the α-chain of the IL-2 receptor, is highly expressed on Tregs, which cause IL-2 deprivation and thus effector T cells death (109); (4) modification of the Foxp3 gene to enhance Treg stability (110–112); and (5) metabolic shifts in Treg from glycolysis to oxidative phosphorylation to enhance anti-inflammatory properties (113, 114). Many studies have shown that Tregs significantly inhibit inflammation and attenuate septic lung injury (115, 116). In the acute phase of sepsis, high-mobility group box 1 protein (HMGB1)-induced activation of chromosome ten (PTEN) reduced TGF-β release from macrophages, leading to decreased Treg differentiation (117). Neutralization of HMGB1 or knockdown of PTEN in macrophages promoted Tregs differentiation and inhibits Th17 differentiation, which alleviated the inflammatory response (117). In addition, Chinese medicinal preparations, such as Berberine and Tanshinone IIA, can inhibit septic inflammation by promoting Tregs differentiation (118, 119). The above studies confirmed that a high proportion of Treg contributed to the abatement of inflammation in septic mice.

However, Treg exacerbated the long-term death of sepsis. Patients and experimental mice that survived sepsis had more significant amounts of Tregs and higher concentrations of IL-33 (74, 120, 121). IL-33, present in the nucleus as a nuclear factor, is rapidly released during cell stress or damage (122). IL-33, in combination with ST2, induces the production of inflammatory factors by DC, macrophages, Th2, Tregs, mast cells, and innate lymphoid cell type 2 (ILC2) (123, 124), promotes the proliferation of Tregs and enhances the inhibitory capacity of Tregs (125, 126). The IL-33/ST2 signaling pathway mediates Treg amplification and exerts immunosuppressive effects. High levels of IL-33 have been found in survivors of sepsis, which in part contributes to a state of persistent immune paralysis. This leads to a depressed immune response and a much higher probability of infection, when the host is re-invaded by the similar or different pathogens. In addition, in a colitis model, IL-33/ST2 signaling was confirmed that increased the frequency of Tregs and ILC2 (127). Siede et al. demonstrated that IL-33/ST2 signaling promotes the expression of TGF-β, IL-10, and Th2-associated cytokines (IL-5/IL-13), and negatively affects effector T cells through the contact-independent mechanism (126). Neutralization of IL-33 significantly inhibited the expansion of Tregs and reduced mortality in mice affected by secondary infection (74). Moreover, most Tregs circulating in the blood originate from the spleen (128). In the immunosuppressive stage, removal of the spleen or use of drugs (e.g., granulocyte-macrophage colony-stimulating factor (GM-CSF), Poria cocos polysaccharides) to reduce the proliferation of Tregs can improve the survival rate and attenuate organ damage in septic mice (129–131). These results reflect the opposite and conflicting effects of Tregs in different stages of sepsis.

Briefly, there are differences in the function and expression levels of Th17 and Tregs in patients with sepsis. The balance of Th17 and Tregs in the body is highly relevant to the prognosis of patients with sepsis. A prospective observational study found heavily reduced numbers of circulating lymphocytes and inverted Th17/Treg ratios in patients with sepsis (34). Gupta et al. also found a high Treg proportion and a low Th17 proportion in post-traumatic sepsis patients, with a significant imbalance of Th17/Treg in cell-mediated immune response and disturbance (41). Zhou et al. found that the level of Th17/Treg ratio was higher in patients with sepsis-induced acute kidney injury (AKI) than in patients without it. Moreover, they confirmed that Th17/Treg imbalance was associated with the occurrence and severity of AKI in sepsis patients (132). Another study has demonstrated a strong positive correlation between the Th17/Treg ratio and the SOFA score: the higher the Th17/Treg ratio, the higher the SOFA score and the worse the prognosis of the patient (41). In a prospective clinical trial, septic patients who initiated enteral nutrition within 48 h had a significantly shorter duration of mechanical ventilation and the ICU hospitalization duration, which was shown to be achieved through modulating Th17/Treg imbalance and inhibiting the IL-23/IL-17 axis (133). Therefore, Th17/Treg imbalance is an essential factor in the pathogenesis of sepsis. Regulating Th17/Treg balance can effectively treat sepsis.

Meanwhile, many animal studies have validated the effect of Th17/Treg imbalance in cecal ligation and puncture (CLP) models of sepsis. It was confirmed that Th17/Treg imbalance caused organ damage and negatively affected survival in septic mice (134–141). Decreased macrophage recruitment and Th17/Treg imbalance were observed in the low-lethality non-severe sepsis model (134). The novel cytokine Metrnl administration reduced the peritoneal bacterial load while improving survival by promoting peritoneal macrophage recruitment and restoring Th17/Treg balance (135). Calcitriol treatment resulted in a more rational Th17/Treg balance in septic obese mice and activation of the renin-angiotensin system (RAS) anti-inflammatory pathway to attenuate acute lung injury (ALI) (136, 137). Nadeem et al. demonstrated that ALI in septic mice was attenuated via modulation of the Th17/Treg immune response and reduction of oxidative stress (138). Restoration of Th17/Treg balance also alleviated lung capillary leak in septic mice (139). Administration of arginine maintained Th17/Treg homeostasis and attenuated hepatic inflammation in sepsis (140). Mesenchymal stem cell (MSC) controlled sepsis-induced inflammatory responses by regulating Th17/Treg balance, which reduced tissue damage, protected organ function, and ultimately improved the survival rate in aged septic rats (141). Luo et al. also demonstrated that MSC modulated the Th17/Treg balance by decreasing IL-17 and RORγt levels and inducing IL-10 and Fxop3 expression via the Gal/Tim-3 pathway (142). As a result, modulation of Th17/Treg balance by various means in the CLP model significantly improved the prognosis of septic mice. This provides strong evidence for further clinical studies. However, the reports of clinical trials of related measures are fewer at present.

## Th17/Treg instability and plasticity

Under physiological conditions, CD4^+^ T cells can differentiate into multiple subtypes, such as Th1, Th2, Th9, Tfh, Th17, Treg, and other T-cell subtypes, which play corresponding immune functions (142). Differing from most somatic cells, T cells remain interconvertible between their subtypes due to instability and plasticity (143–145). Among all Th cell subtypes, Th17 and Treg were considered to be the most plastic subtypes (146, 147). Under appropriate cytokine stimulation or pathogenic conditions, Th17, which exerts a pro-inflammatory effect, can be converted to Treg, suppressing the immune response (148, 149). Jian and his colleagues obtained some Evidence from *in vitro* experiments. They stimulated tumor-infiltrating T lymphocytes (TILs) with OKT3 and mononuclear cells to acquire Th17 clones. After amplifying this subpopulation three times, Foxp3+ cells grew remarkably, while IL-17^+^ cells decreased (150), indicating partial transformation of Th17 to Treg. They are currently thought to result from epigenetic alterations and gene expression profiles reprogramming (148, 150).

In addition, due to prolonged exposure to a pro-inflammatory environment, Treg undergoes Foxp3 expression quiescence and is reprogrammed to Th17-like Treg, exerting pro-inflammatory effects and losing its inhibitory function (151). IL-1β and IL-6 stimulation increase the expression of STAT3 and RORγt in the Treg. This promotes the conversion of Treg to Th17-like Treg, releasing pro-inflammatory IL-17A (152). IL-23 exacerbates this phenomenon (152). TGF-β serves as a key factor in inducing Treg differentiation. Yang et al. used TGF-β to prompt Foxp3 expression. Then intervention with IL-6 was performed, and the results showed that the expression of Foxp3 was significantly downregulated, while the expression of IL-17 was increased (153). Similarly, inhibition of indoleamine 2,3-dioxygenase (IDO), a plasmacytoid DC-produced tryptophan-catabolizing enzyme that maintains Treg/Th17 balance, increased IL-6 expression and promoted the transformation of Treg into Th17 (154). Another study has shown that using single-cell RNA sequencing to focus on T cell subtype alteration in chronically exposed hypercholesterolemic environments (155). The results confirmed the continuous increase of Treg in atherosclerotic mice, which were partially transformed into Th17-like cells with highly pro-inflammatory properties (155). These findings are indicative of the instability and plasticity between Th17 and Treg, which are capable of transforming into each other.

Reports on Treg in sepsis are mixed. Some studies have demonstrated that increased circulating Treg proportion contributed to lymphocyte dysfunction and significantly reduced survival (156), while suppression of Treg percentage improved survival in sepsis (157). In contrast, other studies have found that adoptive transfer of Treg or pharmacologic modulation of Treg response protected septic mice from death (158, 159). Apart from the excessive inflammatory response and immune paralysis associated with sepsis, the underlying cause of this conflicting result may lie in the dysregulation of plasticity between Treg and Th17, leading to different immune effects of Treg.

## Th17/Treg balance in different stages of sepsis

Sepsis undergoes a sustained initial phase of hyperinflammation followed by a protracted period of immunosuppression (72, 160). Patients surviving sepsis often die from secondary infections due to immunosuppression and organ damage (161). Uncontrolled lymphocyte apoptosis is thought to be the major cause of immunosuppression (10, 160). As stated by Cao et al., sepsis is a life-and-death race between the pathogen and the host immune response, and the competition between the two determines the prognosis of septic patients (160, 162). In this process, Th17 and Tregs’ effects change during sepsis.

In the acute phase of sepsis, the proportion of Th17 is markedly elevated in the peripheral blood of the host, accompanied by an expansion in Tregs (130, 132). Invading bacteria activate T cell function, induce Th17 differentiation, and trigger an adaptive immune response to clear the bacteria (163, 164). However, persistent infection leads to excessive inflammatory activation and promotes Treg differentiation to avoid organ damage from hyper-inflammation (15, 165). Studies have shown that patients suffering from sepsis have an increased percentage of Tregs in the peripheral blood, which occurs mainly in the early stages of sepsis. Persistently high Treg counts are a vital factor in the poor prognosis of patients (38, 166). Furthermore, it has been verified in a sepsis mouse model that Treg percentage showed a significant augmentation following CLP, a process that lasted up to 7 days (167). Control of Tregs and Th17 ratio and maintenance of Th17/Treg balance can effectively affect the prognosis of sepsis. Lu et al. discovered that mucosa-associated lymphoid tissue lymphoma translocation protein 1 (MALT1) overexpression promoted the polarization of CD4^+^ T cells towards Th17 and increased the Th17/Treg ratio, which in turn promoted systemic inflammation, elevated levels of oxidative stress, and exacerbated organ damage (168). Meanwhile, receiving MSC intravenous treatment, septic mice showed a lower Th17/Treg ratio in peripheral blood and spleen, protecting organs from inflammatory damage and increasing the 72 h survival rate (140). However, another study has reported that increasing the intestinal Th17 population reduced intestinal inflammation and bacterial translocation, protected mice from endotoxemia-induced intestinal injury, and enhanced survival (169). This may be a hyperacute phase response triggered when intestinal bacteria invade the host, thus helping the host eliminate the bacteria before they enter the bloodstream in large numbers, avoiding a storm of inflammatory factors. Meanwhile, it cannot be ruled out that due to Th17 plasticity, pathogen stimulation and alteration of the cytokine environment promoted the reprogramming of Th17 to Treg, which suppressed the inflammatory response in the gut (148, 149). However, more experiments are needed to clarify. The above study confirmed that the ratio of Th17/Treg dramatized the prognosis of sepsis. In the early phase of sepsis, excessive inflammatory response causes organ damage. Increasing the proportion of Tregs, decreasing the proportion of Th17, and correcting the Th17/Treg balance are essential factors for restoring immune homeostasis, reducing inflammatory organ damage, and decreasing mortality.

When pathogens are effectively cleared, immune balance can be restored; conversely, immune regulation is imbalanced, anti-inflammatory responses are enhanced, and the host is susceptible to a state of immunosuppression or even organ failure (58). In the later stages of sepsis, the proportion of Treg increased, accompanied by a decrease in the proportion of Th17, which led to a lower Th17/Treg ratio (34). This was related to the initial inflammatory factor storm causing inflammatory factor depletion and severe T cell apoptosis (34). Lymphopenia coupled with increased apoptosis and decreased proliferation of lymphocytes, and more circulating Treg are associated with persistent organ dysfunction, secondary infections and long-term mortality (170, 171). Therefore, in the late stage of sepsis, restoring T cell function and promoting inflammatory response can help enhance host immunity and effectively avoid immunosuppression, reducing the late mortality of sepsis patients. In a two-stroke model of sepsis, methicillin-resistant Staphylococcus aureus (MRSA) inoculation was used to trigger secondary pneumonia (172). The presence of long-term immune dysfunction in septic mice was confirmed by higher bacterial counts in bronchoalveolar lavages, spleen and kidney homogenates, increased T-cell apoptosis, enhanced Treg ratio, accompanied by severe lung injury, and a markedly higher 20-day mortality rate (172). Treatment with citrulline restored T-cell mitochondrial activity, reduced the number of Tregs, and reversed the mortality rate in mice (172). In addition, during the immunosuppressive phase of sepsis, IL-33, GM-CSF or tumor necrosis factor receptor antibody (DTA-1) administration can inhibit Treg amplification and restore Th17/Treg balance, favoring the survival of sepsis (120, 130, 173).

Currently, many studies are exploring the mechanisms of Th17/Treg imbalance at different stages of sepsis. In the early stage, pathogens invade the organism and activate innate immune cells to phagocytose and clear them, triggering the innate immune response and complement system to produce various pro-inflammatory factors, e.g., IL-1β, IL-6, IL-12, and TNF-α (174, 175). The study has demonstrated that complement C5a induced DC transfer from the peritoneal cavity to peripheral blood and lymph nodes, inducing the expansion Th17 (176). Co-incubation of human DC with gram-positive bacteria cell wall peptidoglycan polymers stimulates the production of IL-23 and IL-1β by DC, thereby inducing the CD4^+^ T cells to differentiate to Th17 (177). Inflammatory factors stimulate T-lymphocyte proliferation and differentiation, increasing the proportion of Th17. The persistently increased Th17 continues to secrete pro-inflammatory cytokines, further inducing cytokine storm and Th17/Treg imbalance. Furthermore, most patients who survive sepsis develop a prolonged immunocompromised state, which is mainly caused by immunosuppression in the late stage of sepsis (178, 179). The excessive inflammatory response prompts overexpression of immune checkpoint molecules (e.g., PD-1, TIM-3, CTLA-4) (58). These negative co-stimulatory factors cause Th17 decrease and T-cell depletion by inhibiting T-lymphocyte activation and proliferation or by inducing apoptosis (180–183). The relative increase in Treg during sepsis was shown to be due to the high resistance of Treg to apoptosis and preferential loss of other subtypes (Th1, Th2, Th17, Tfh), which ultimately leads to a Th17/Treg balance toward Treg (156, 184–187). In addition, inflammation-related markers are also associated with Treg amplification. C3aR/C5aR-mediated inflammatory activity impairs Treg function (188). Excessive inflammatory response in the sepsis leads to complement C3a depletion, which is thought to be associated with Treg amplification (189). Administration of exogenous complement C3a inhibited Treg expansion in sepsis animal model (190). In addition, cytokine IL-33 also upregulates Treg and contributes to sepsis-induced immunosuppression by promoting M2 macrophage polarization and IL-10 secretion (74). The above pathways increase the Treg proportion in late spies, inducing Th17/Treg balance biased toward Treg.

However, the great instability and plasticity of Th17/Treg left multiple possibilities for immune alterations in the septic process. Under certain conditions, Th17 and Treg underwent functional changes through internal reprogramming to express other T-cell related cytokines. Th17 can be transformed into Th1, Th2, Treg, and Tfh. In contrast, Treg can also be transformed into Th1-like, Th2-like, and Th17-like, Tfh-like Treg, which was confirmed in many studies in the tumor microenvironment, autoimmune disease, allergic asthma, and multiple sclerosis (MS) (90, 146, 191–197). Both Th17 and Treg required TGF-β to regulate their differentiation (90, 198). Treg inhibited cell differentiation into Th17 by inhibiting RORyt activity through Foxp3 expression (22). This restriction can be lifted by IL-6 and other cytokines, and undergone reprogramming of Th17 and Treg (22). In the early stages of sepsis, the Th17/Treg balance favored Th17 in the presence of pathogen stimulation and a strong inflammatory microenvironment. However, there was still no evidence to confirm whether Treg lost its suppressive function or reprogrammed to Th17, further promoting a hyperinflammatory response. Similarly, in advanced sepsis, both Th17 and Tregs showed decreased numbers due to lymphocyte apoptosis. Th17/Treg favored Treg, and showed an overall immunosuppressive state. Nevertheless, whether this state was due to the plasticity and instability of Th17 leading to the transformation of Th17 to Treg remained unknown in the field of sepsis research. Overall, it can be concluded that the Th17/Treg balance has diametrically opposing effects on host prognosis at different stages of sepsis, and the underlying phenotypic and functional alterations were unclear.

## Th17/Treg balance in different organs of sepsis

Most studies have focused on the function and characterization of Th17/Treg in the circulation or spleen (34, 41, 132, 133, 137). However, Th17 and Treg are prevalent not only within the immune organs but also in the lungs, kidneys, liver, heart, brain, and muscle (199–204). The pathophysiological features of sepsis vary from organ to organ due to the functional and structural differences of each organ. Th17/Treg balance has tissue characterization on each organ.

The lung is the most susceptible and is the first organ to be engaged in sepsis (205). Sepsis often causes pulmonary inflammation that fails to subside, inducing ALI/acute respiratory distress syndrome (ARDS) with a high morbidity and mortality rate (206, 207). The pathology is characterized primarily by a massive inflammatory cell infiltrate, loss of barrier integrity and increased permeability of alveolar capillaries, leading to tissue damage in inflammation (207, 208). Many studies have shown that restoration of Th17/Treg balance has a positive effect on sepsis-induced lung injury (137, 138, 209, 210). Xia et al. demonstrated that Maresin 1, an emerging specific pro-inflammatory mediator, increased the number of Tregs and decreased the Th17 count, which regulated the Th17/Treg balance (210). Altered T-cell balance significantly inhibited excessive inflammatory response, promoted inflammatory regression, reduced septic lung damage and improved lung function (210). On the other hand, Nadeem et al. also acted to regulate the Th17/Treg balance by inhibiting the expression of ITK, an essential regulator of Th17 differentiation, reducing Th17 and expanding Tregs (137). This behavior reversed the level of airway inflammation and oxidative stress in septic lungs (137). In addition, Traditional Chinese medicine (TCM) also played a positive role in modulating Th17/Treg balance. Our previous study confirmed that berberine attenuated pulmonary edema and hypoxemia in septic mice by regulating Th17/Treg homeostatic (209). Paclitaxel also alleviated lung injury and prolonged survival in septic mice by altering the Th17 and Treg populations.

Besides the lungs, the kidneys are often vulnerable to sepsis. Many studies have focused on the interaction of Th17 or Treg with sepsis-induced acute kidney injury (SAKI) (211–214). A high level of IL-17 and tissue infiltration of Th17 often accompanies SAKI (215). Inhibition of Th17 differentiation and function can effectively alleviate renal damage (216). Additionally, several animal studies confirmed that Tregs protected the kidney from inflammation (214, 217, 218). However, other studies suggested that Treg exacerbated renal damage, which could be attenuated by depletion of Treg and inhibition of IL-10 (219, 220). This called for consideration of the impact of Treg plasticity on SAKI during different phases of sepsis. Alterations in the phenotype and function of Treg can partially contribute to the inflammatory response towards the two opposite outcomes. Treg exerting anti-inflammatory effects effectively alleviates renal inflammation in SAKI (213). Nevertheless, Treg with pro-inflammatory properties, such as Th17-like Treg, can exacerbate immunopathologic changes in the kidney, but further studies were necessary to explain this. Furthermore, contradictory results encouraged us to concentrate on the impact of Th17/Treg balance on SAKI. Zhou et al. investigated Th17/Treg balance and found that patients with SAKI had an elevated Th17 ratio and Th17/Treg imbalance (132). The Th17/Treg balance axis tended to favor the Th17 lineage (132). The Th17/Treg ratio was an independent risk factor for SAKI (132). Therefore, perhaps it is not Th17 or Tregs, but rather the Th17/Treg balance that influenced the pathological process of SAKI.

## Discussion

Variation in the immune function affects regression in septic patients. The differentiation and function of Th17 and Treg, and bias in Th17/Treg balance, determine different grades of inflammatory response and organ damage. Many studies are still limited to pathophysiological alterations of Th17 or Treg in sepsis, along with conflicting results. This forced us to focus on Th17/Treg balance rather than solely on Th17 or Treg. However, the current established studies have limited insights into Th17/Treg balance in sepsis. This review summarizes the pathophysiology of Th17 and Treg in sepsis, and describes the effects of Th17/Treg balance in different inflammatory stages and organs. Since Th17/Treg balance controls the shifts in pro-inflammatory and anti-inflammatory responses, amelioration of sepsis by clarifying the optimal point of Th17/Treg balance may be an effective therapeutic intervention.

## Author contributions

XL: Funding acquisition, Investigation, Resources, Visualization, Writing – original draft, Writing – review & editing. LC: Funding acquisition, Investigation, Resources, Visualization, Writing – original draft, Writing – review & editing. WP: Data curation, Visualization, Writing – original draft. HD: Data curation, Visualization, Writing – original draft. HN: Data curation, Visualization, Writing – original draft. HT: Data curation, Visualization, Writing – original draft. HH: Data curation, Visualization, Writing – original draft. SW: Data curation, Visualization, Writing – original draft. JQ: Data curation, Visualization, Writing – original draft. AL: Conceptualization, Project administration, Visualization, Writing – review & editing. KC: Conceptualization, Funding acquisition, Visualization, Writing – review & editing.
